# Supplementary material for: Evidence on strategies promoting discharge planning participation in patients with chronic heart failure: A scoping review protocol
Source: PLoS One. 2026 May 14;21(5):e0348367. doi: 10.1371/journal.pone.0348367 (PMC13175467; doi:10.1371/journal.pone.0348367)
Supplement: Attachment — Submitted filename: Response to Reviewers.docx [file pone.0348367.s004.docx]

**Response to Reviewers**

**Manuscript ID:** PONE-D-25-67198

**Title:** Evidence on strategies promoting discharge planning participation in patients with chronic heart failure: A scoping review protocol

[March 28, 2026]

Dear Dr. Divya Bhandari and Reviewers,

We thank the Academic Editor and both reviewers for their thorough and constructive evaluation of our manuscript. Your insightful comments have helped us to substantially improve the clarity, methodological rigor, and transparency of this protocol.

We have carefully considered all points raised and have revised the manuscript accordingly. Below, we provide a point-by-point response to each comment. All changes in the revised manuscript are highlighted using track changes for ease of reference.

**Journal Requirements**

**Requirement 1: PLOS ONE Style and Formatting**

**Editor comment:** Please ensure that your manuscript meets PLOS ONE's style requirements, including those for file naming.

**Response:** We have carefully reviewed the PLOS ONE formatting guidelines and revised the manuscript to ensure full compliance, including file naming conventions, heading styles, and reference formatting.

**Requirement 2: ORCID iD for Corresponding Author**

**Editor comment:** PLOS requires an ORCID iD for the corresponding author in Editorial Manager on papers submitted after December 6th, 2016.

**Response:** The corresponding author has confirmed that a validated ORCID iD is linked in Editorial Manager.

**Requirement 3: Evaluation of Cited References**

**Editor comment:** If the reviewer comments include a recommendation to cite specific previously published works, please review and evaluate these publications.

**Response:** We have carefully evaluated all publications recommended by the reviewers and incorporated those deemed relevant into the revised manuscript with appropriate citations. Specifically, we have taken into account the methodological insights of PAGER mentioned by Reviewer 1, which are helpful for enhancing the transparency of our research, and relevant citations have been added accordingly.

**Academic Editor Comments**

The Academic Editor noted that Reviewer #2's concern about the manuscript being presented as a protocol rather than a completed study does not require action, as PLOS ONE publishes study protocols. The Editor encouraged us to address as many of Reviewer 1's comments as feasible and to ensure the manuscript clearly adheres to PLOS ONE protocol submission guidelines. We thank the Editor for this clarification and have followed this guidance throughout our revision.

**Reviewer #1 Comments**

We sincerely thank Reviewer #1 for the detailed and constructive feedback. We have addressed each major and minor comment below.

**Major Comment 1: Search strategy – clarification needed**

**Reviewer #1:** Although several databases were included, it is unclear whether the search strategy was developed with input from a health sciences librarian or information specialist. Given best practice for scoping and systematic reviews, please clarify whether librarian input was sought. If not, consider engaging a librarian and documenting their involvement.

**Response:** Thank you for this valuable suggestion regarding search strategy development in scoping reviews. We acknowledge that the initial search strategy was developed by the research team without formal input from a health sciences librarian. In response to your comment, we have consulted a health sciences librarian from our university to review and refine the search strategy, and we have updated the manuscript to document this involvement. The revised search strategy maintains the original scope while improving its comprehensiveness and transparency.

**Manuscript change:**

**Major Comment 2: Definition of patient participation – needs alignment with inclusion criteria**

**Reviewer #1:** These two conceptualizations misalign. I recommend revising the Introduction to reflect the broader definition used in the Methods. The decision‑making‑centric conceptualization is now quite outdated and grounded in a traditional medical model; contemporary frameworks describe participation as multidimensional and wide‑ranging.

**Response:** We agree with this observation and thank the reviewer for the precise identification of this conceptual inconsistency. In the revised Introduction, we have replaced the earlier decision-making-centric definition with a broader, multidimensional conceptualization of patient participation. This updated definition is now consistent with the inclusion criteria and with contemporary frameworks cited in the literature.

**Manuscript change:**

**Major Comment 3: Thematic narrative analysis – require methodological clarity**

**Reviewer #1:** Although you state that the review will use **thematic narrative analysis**, the protocol does not specify:

* which methodological framework or reference underpins this analysis, and

* the detailed steps you will follow.

Please add a citation for the methodological approach and outline your analytic process clearly. As this is a protocol, transparency is essential so readers can later evaluate the consistency between the planned and completed review.

**Response:** We thank the reviewer for this important point. We have now clearly specified in the Data Synthesis section that thematic synthesis will follow the three-stage framework described by Thomas and Harden, which involves: (1) line-by-line coding of extracted data by two independent reviewers; (2) development of descriptive themes reflecting the content of included studies; and (3) generation of analytical themes by interpreting descriptive themes against the conceptual definition of patient participation.

**Manuscript change:**

**Major Comment 4: Research Question #3 – clarify approach to “mapping evidence gaps”**

**Reviewer #1:** Research Question #3 relates to identifying gaps in the evidence base, but the process for achieving this is not fully described.

As phrased, this could be mistaken for *evidence gap mapping*, a distinct methodology.

Consider clarifying:

* how you will identify and present gaps within the scoping review framework, and

* whether you will use a structured approach such as the **PAGER framework** (Bradbury-Jones et al., 2022) to support the reporting and interpretation of gaps. This would strengthen coherence with your stated aims.

**Response:** We thank the reviewer for this helpful clarification. Research Question #3 does not employ evidence gap mapping as a distinct methodology; rather, gap identification is embedded within the scoping review process through the application of the PAGER framework. We have revised the Methods section to make this explicit. Specifically, we now state that the PAGER framework (Patterns, Advances, Gaps, Evidence for Practice, and Research Recommendations) will be used to structure the presentation of findings, including the identification of evidence-practice gaps. Each PAGER component within the context of this review was defined operationally in Table 2 of the revised manuscript.

**Manuscript change:**

**Major Comment 5: Alignment with Levac et al. – restructure for clarity**

**Reviewer #1:** Although you refer to Levac et al. (2010), the Methods section does not clearly map onto their six-stage framework:

1. Identifying the research question

2. Identifying relevant studies

3. Selecting studies

4. Charting the data

5. Collating, summarizing, and reporting the results

6. Consulting with stakeholders

To improve transparency and methodological alignment, consider:

* **renaming subheadings** to reflect these stages, or

* **explicitly stating** within each section which Levac step is being addressed.

For example:

* *Research Questions - Step 1: Identifying the Research Question**

* *Search Strategy - Step 2: Identifying Relevant Studies**

Also, **Step 6 (Consultation with Stakeholders)** is currently absent and should be added or justified.

**Response:** We appreciate this structural feedback. We have revised the Methods section to align each part with the corresponding stage of the Levac et al. framework by using the stage labels as section headings. Regarding Stage 6: Consultation, this will be conducted after completion of the review. Findings will be shared with relevant clinical and policy stakeholders to gather input on practice implications. This feedback will help validate the findings, refine evidence–practice gaps, and strengthen recommendations. This stage is intended to enhance the relevance of the review and is not part of the formal data analysis.

**Manuscript change:**

**Minor Comment 1: Date Limits Justification**

**Reviewer #1:** If date limits are applied to the search, please briefly justify them.

**Response:** We have clarified in the revised manuscript that no date restrictions will be applied to the literature search; the specific search period will be determined when the study is formally initiated.

**Manuscript change:**

**Minor Comment 2: Consistent Use of Terminology**

**Reviewer #1:** Ensure consistent use of terminology (e.g., participation, engagement, involvement) once you revise the conceptual framing.

**Response:** We have systematically reviewed the entire manuscript and standardized the use of terminology. "Patient participation" is now used as the primary term throughout, consistent with the revised conceptual definition. Where "engagement" or "involvement" appears, it is used either as a component of participation construct or in the context of a direct citation. Track changes in the revised manuscript reflect all instances where terminology has been updated.

**Manuscript change:**

**Reviewer #2 Comments**

**Reviewer #2:** After review, I found that the manuscript is presented more as a protocol or proposal rather than a complete research paper, as it does not include full findings and discussion. The title and topic are strong and promising, especially for advancing patient‑centered decision making, but the work needs to be developed into a full article before it can be considered for publication.

**Response:** We thank Reviewer #2 for their positive comments regarding the topic and title. We respectfully note, as clarified by the Academic Editor, that PLOS ONE does publish study protocols and that this submission is intentionally formatted as a protocol rather than a completed study. The manuscript clearly identifies itself as a scoping review protocol throughout, including in the title, abstract, and Methods section. We have ensured that all required elements for protocol submissions outlined in the PLOS ONE guidelines are fully detailed in the revised version, including the registered OSF protocol link (https://doi.org/10.17605/OSF.IO/QPAZM), the inclusion/exclusion criteria, the data extraction process, and the planned analytical approach.

We hope that these revisions adequately address all concerns raised. We remain available to provide any further clarification or to make additional revisions as required. We look forward to the Editor's decision.

Sincerely,

Shouqing Lin (Corresponding Author)

Email: y51617@126.com

Department of Obstetrics and Gynecology, Peking Union Medical College Hospital, Chinese Academy of Medical Sciences & Peking Union Medical College, Beijing, China.

On behalf of all authors
